# *N*-(4-Bromo­phen­yl)acetamide: a new polymorph

**DOI:** 10.1107/S1600536813005448

**Published:** 2013-02-28

**Authors:** Jerry P. Jasinski, Curtis J. Guild, H. S. Yathirajan, B. Narayana, S. Samshuddin

**Affiliations:** a Department of Chemistry, Keene State College, 229 Main Street, Keene, NH 03435-2001, USA; b Department of Studies in Chemistry, University of Mysore, Manasagangotri, Mysore 570 006, India; c Department of Studies in Chemistry, Mangalore University, Mangalagangotri, 574 199, India

## Abstract

A new polymorph of the title compound, C_8_H_8_BrNO, has been determined at 173 K in the space group *P*2_1_/*c*. The previous room-temperature structure was reported to crystallize in the ortho­rhom­bic space group *Pna*2_1_ [Andreetti *et al.* (1968 ▶). *Acta Cryst.* B**24**, 1195–1198]. In the crystal, mol­ecules are linked by N—H⋯O hydrogen bonds forming chains along [010]. Weak C—H⋯π inter­actions are also present.

## Related literature

For 2-aryl­acetamides, see: Mijin & Marinkovic (2006 ▶); Mijin *et al.* (2008 ▶) and for amides, see: Wu *et al.* (2008 ▶, 2010 ▶). For the structure of the ortho­rhom­bic polymorph, see: Andreetti *et al.* (1968 ▶). For related structures, see: Praveen *et al.* (2011*a* ▶,*b* ▶,*c* ▶). For standard bond lengths, see: Allen *et al.* (1987 ▶).
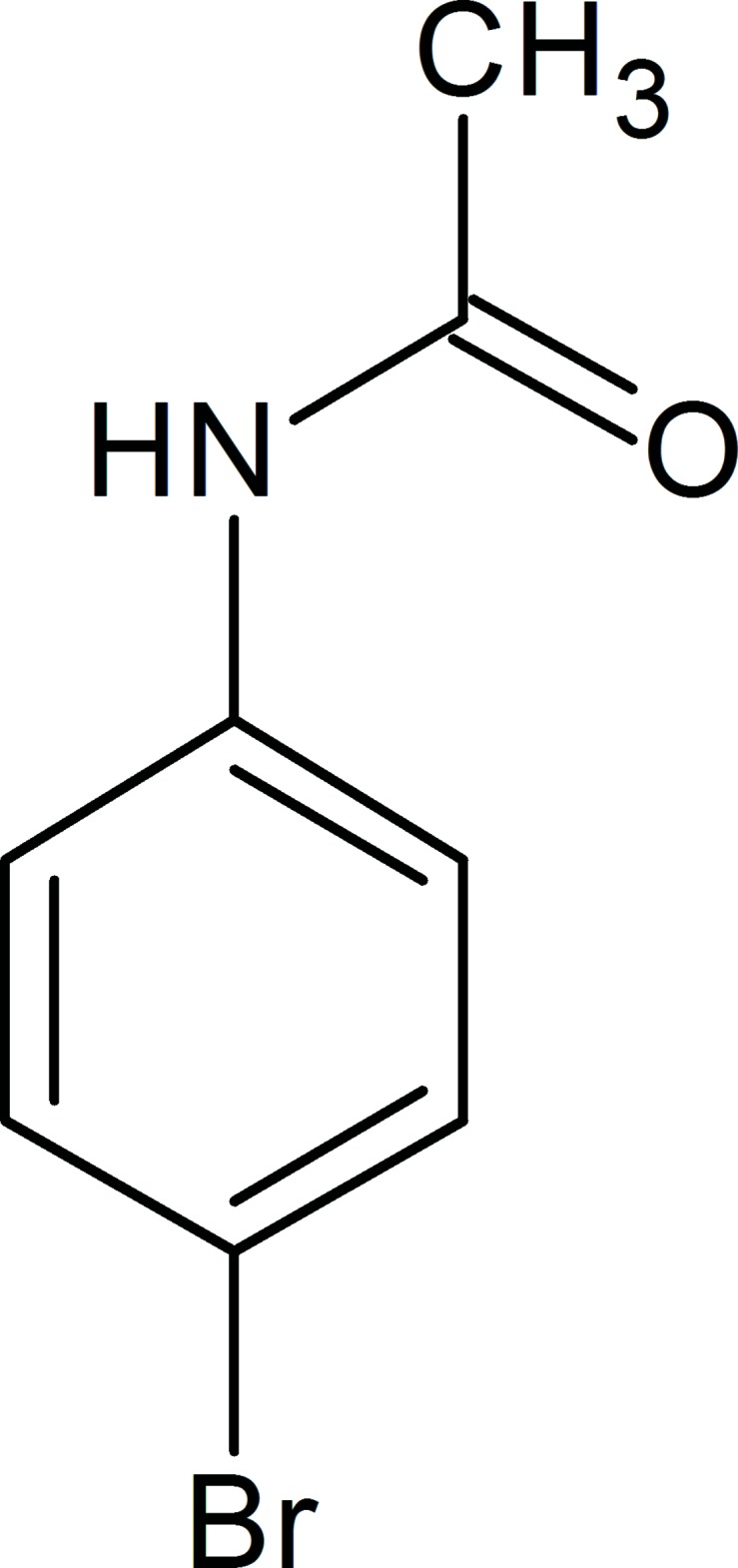


## Experimental

### 

#### Crystal data

C_8_H_8_BrNO
*M* *_r_* = 214.06
Monoclinic, 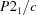

*a* = 6.7250 (7) Å
*b* = 9.3876 (11) Å
*c* = 14.4434 (14) Å
β = 117.750 (4)°
*V* = 806.96 (15) Å^3^
*Z* = 4
Mo *K*α radiation
μ = 5.03 mm^−1^
*T* = 173 K
0.32 × 0.22 × 0.18 mm

#### Data collection

Oxford Diffraction Xcalibur (Eos, Gemini) diffractometer
Absorption correction: multi-scan (*CrysAlis PRO*; Oxford Diffraction 2010 ▶) *T*_min_ = 0.296, *T*_max_ = 0.465
10902 measured reflections
2689 independent reflections
2099 reflections with *I* > 2σ(*I*)
*R*_int_ = 0.035

#### Refinement

*R*[*F* ^2^ > 2σ(*F* ^2^)] = 0.033
*wR*(*F* ^2^) = 0.073
*S* = 1.04
2689 reflections
101 parameters
H-atom parameters constrained
Δρ_max_ = 0.54 e Å^−3^
Δρ_min_ = −0.44 e Å^−3^

### 

Data collection: *CrysAlis PRO* (Oxford Diffraction, 2010 ▶); cell refinement: *CrysAlis PRO*; data reduction: *CrysAlis RED* (Oxford Diffraction, 2010 ▶); program(s) used to solve structure: *SHELXS97* (Sheldrick, 2008 ▶); program(s) used to refine structure: *SHELXL97* (Sheldrick, 2008 ▶); molecular graphics: *SHELXTL* (Sheldrick, 2008 ▶); software used to prepare material for publication: *SHELXTL*.

## Supplementary Material

Crystal structure: contains datablock(s) global, I. DOI: 10.1107/S1600536813005448/su2566sup1.cif

Structure factors: contains datablock(s) I. DOI: 10.1107/S1600536813005448/su2566Isup2.hkl

Supplementary material file. DOI: 10.1107/S1600536813005448/su2566Isup3.cml

Additional supplementary materials:  crystallographic information; 3D view; checkCIF report

## Figures and Tables

**Table 1:** Hydrogen-bond geometry (Å, °) *Cg*1 is the centroid of the C3–C8 ring.

*D*—H⋯*A*	*D*—H	H⋯*A*	*D*⋯*A*	*D*—H⋯*A*
N1—H1⋯O1^i^	0.89	2.00	2.885 (2)	174
C1—H1*B*⋯*Cg*1^ii^	0.98	2.84	3.761 (3)	157

Symmetry codes: (i) 
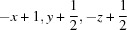
; (ii) 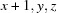.

**Fig. 1.**
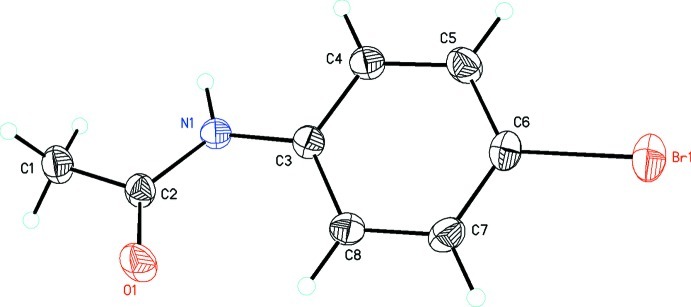
The molecular structure of the title molecule, showing the atom labelling. Displacement ellipsoids are drawn at the 50% probability level.

**Fig. 2.**
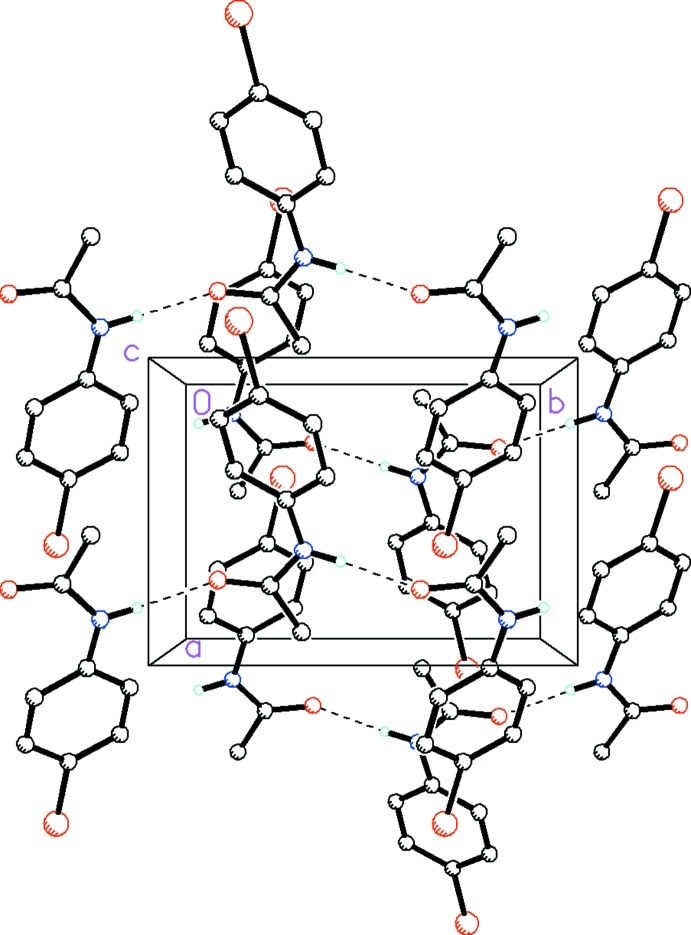
The crystal packing diagram of the title compound viewed along the c axis. Dashed lines indicate N—H···O hydrogen bonds linking the molecules into chains along [010]. C-bound H atoms have been omitted for clarity.

## supplementary crystallographic information

### Comment

Substituted 2-arylacetamides are very interesting compounds because of their structural similarity to the lateral chain of natural benzyl penicillin (Mijin *et al.*, 2006, 2008). Amides are also used as ligands due to their excellent coordination abilities (Wu *et al.*, 2008, 2010). The room temperature crystal structure of the title compound was reported without hydrogen atom coordinates in the orthorhombic space group Pna2_1_ (Andreetti *et al.*, 1968). We report herein on the crystal structure of the monoclinic polymorph that crystallized in space group P2_1_/c.

The molecular structure of the title compound is illustrated in Fig. 1. Bond lengths are in normal ranges (Allen *et al.*, 1987) but show slight changes from those reported for some similar acetamide derivatives *viz*., *N*-(4-chloro-1,3-benzothiazol-2-yl)-2-(3-methylphenyl)acetamide monohydrate (Praveen *et al.*, 2011*a*), *N*-(3-chloro-4-fluorophenyl)-2,2-diphenylacetamide (Praveen *et al.*, 2011*b*) and *N*-(3-chloro-4-fluorophenyl)-2-(naphthalen-1-yl)acetamide (Praveen *et al.*, 2011*c*). The differences observed are primarily in the acetamide and bromophenyl regions [C1–C2 1.501 (3) Å *versus* 1.53 (4) Å; N1–C2 1.347 (2) Å *versus* 1.30 (3) Å; N1–C3 1.401 (2) Å *versus* 1.44 (3) Å and C6–Br 1.8907 (19) *versus* 1.91 (1) Å].

In the crystal, molecules are linked by N—H···O hydrogen bonds forming chains along [010] [Table 1 and Fig. 2]. Weak C—H···π interactions are also present (Table 1) and contribute to the crystal packing stability.

### Experimental

4-Bromo aniline (0.172 g, 1 mmol) was dissolved in acetic acid (20 ml) and refluxed for 4 h. The solution was then cooled and poured into 100 ml of ice-cold water with stirring. The precipitate obtained was filtered, washed with water and dried. Block-like yellow crystals were grown from a solution in ethyl acetate by slow evaporation of the solvent (M.p.: 430 K).

### Refinement

The NH H atom was located in a difference Fourier map and refined as a riding atom: N-H = 0.89 Å with U_iso_(H) = 1.2U_eq_(N). The C-bound H atoms were placed in calculated positions and refined as riding atoms: C—H = 0.95 Å (CH) and 0.98 Å (CH_3_) with U_iso_(H) = 1.2U_eq_(C) for CH H atoms and = 1.5U_eq_(C) for CH_3_ H atoms.

### Crystal data

**Table:**

C_8_H_8_BrNO	*F*(000) = 424
*M**_r_* = 214.06	*D*_x_ = 1.762 Mg m^−^^3^
Monoclinic, *P*2_1_/*c*	Mo *K*α radiation, λ = 0.71073 Å
Hall symbol: -P 2ybc	Cell parameters from 3269 reflections
*a* = 6.7250 (7) Å	θ = 3.4–32.3°
*b* = 9.3876 (11) Å	µ = 5.03 mm^−^^1^
*c* = 14.4434 (14) Å	*T* = 173 K
β = 117.750 (4)°	Block, yellow
*V* = 806.96 (15) Å^3^	0.32 × 0.22 × 0.18 mm
*Z* = 4	

### Data collection

**Table:**

Oxford Diffraction Xcalibur (Eos, Gemini) diffractometer	2689 independent reflections
Radiation source: Enhance (Mo) X-ray Source	2099 reflections with *I* > 2σ(*I*)
Graphite monochromator	*R*_int_ = 0.035
Detector resolution: 16.1500 pixels mm^-1^	θ_max_ = 32.3°, θ_min_ = 3.4°
ω scans	*h* = −9→9
Absorption correction: multi-scan (*CrysAlis PRO*; Oxford Diffraction 2010)	*k* = −13→13
*T*_min_ = 0.296, *T*_max_ = 0.465	*l* = −20→19
10902 measured reflections	

### Refinement

**Table:**

Refinement on *F*^2^	Primary atom site location: structure-invariant direct methods
Least-squares matrix: full	Secondary atom site location: difference Fourier map
*R*[*F*^2^ > 2σ(*F*^2^)] = 0.033	Hydrogen site location: inferred from neighbouring sites
*wR*(*F*^2^) = 0.073	H-atom parameters constrained
*S* = 1.04	*w* = 1/[σ^2^(*F*_o_^2^) + (0.0329*P*)^2^ + 0.1779*P*] where *P* = (*F*_o_^2^ + 2*F*_c_^2^)/3
2689 reflections	(Δ/σ)_max_ = 0.001
101 parameters	Δρ_max_ = 0.54 e Å^−^^3^
0 restraints	Δρ_min_ = −0.44 e Å^−^^3^

### Special details

**Table:**

Geometry. All e.s.d.'s (except the e.s.d. in the dihedral angle between two l.s. planes) are estimated using the full covariance matrix. The cell e.s.d.'s are taken into account individually in the estimation of e.s.d.'s in distances, angles and torsion angles; correlations between e.s.d.'s in cell parameters are only used when they are defined by crystal symmetry. An approximate (isotropic) treatment of cell e.s.d.'s is used for estimating e.s.d.'s involving l.s. planes.
Refinement. Refinement of *F*^2^ against ALL reflections. The weighted *R*-factor *wR* and goodness of fit *S* are based on *F*^2^, conventional *R*-factors *R* are based on *F*, with *F* set to zero for negative *F*^2^. The threshold expression of *F*^2^ > σ(*F*^2^) is used only for calculating *R*-factors(gt) *etc*. and is not relevant to the choice of reflections for refinement. *R*-factors based on *F*^2^ are statistically about twice as large as those based on *F*, and *R*- factors based on ALL data will be even larger.

### Fractional atomic coordinates and isotropic or equivalent isotropic displacement parameters (Å^2^)

**Table:**

	*x*	*y*	*z*	*U*_iso_*/*U*_eq_	
Br1	−0.12616 (4)	0.70900 (2)	0.493749 (17)	0.03329 (8)	
O1	0.4333 (2)	0.63844 (14)	0.19611 (11)	0.0286 (3)	
N1	0.3872 (3)	0.85329 (15)	0.25584 (12)	0.0212 (3)	
H1	0.4358	0.9432	0.2658	0.025*	
C1	0.5678 (4)	0.8449 (2)	0.14665 (17)	0.0283 (4)	
H1A	0.5162	0.8041	0.0767	0.042*	
H1B	0.7312	0.8344	0.1869	0.042*	
H1C	0.5281	0.9462	0.1404	0.042*	
C2	0.4570 (3)	0.76819 (19)	0.20162 (15)	0.0213 (4)	
C3	0.2696 (3)	0.81333 (18)	0.31030 (14)	0.0199 (3)	
C4	0.2647 (3)	0.91062 (19)	0.38173 (15)	0.0240 (4)	
H4	0.3428	0.9985	0.3931	0.029*	
C5	0.1473 (3)	0.8806 (2)	0.43633 (16)	0.0265 (4)	
H5	0.1435	0.9475	0.4849	0.032*	
C6	0.0351 (3)	0.7520 (2)	0.41948 (15)	0.0233 (4)	
C7	0.0369 (3)	0.6541 (2)	0.34821 (15)	0.0247 (4)	
H7	−0.0407	0.5662	0.3374	0.030*	
C8	0.1522 (3)	0.68544 (19)	0.29313 (15)	0.0235 (4)	
H8	0.1518	0.6195	0.2431	0.028*	

### Atomic displacement parameters (Å^2^)

**Table:**

	*U*^11^	*U*^22^	*U*^33^	*U*^12^	*U*^13^	*U*^23^
Br1	0.03360 (13)	0.03978 (14)	0.03455 (13)	−0.00199 (9)	0.02264 (10)	0.00392 (9)
O1	0.0383 (8)	0.0163 (6)	0.0388 (8)	0.0038 (6)	0.0242 (7)	−0.0003 (5)
N1	0.0257 (8)	0.0132 (7)	0.0290 (8)	−0.0018 (6)	0.0163 (7)	−0.0016 (6)
C1	0.0328 (11)	0.0252 (9)	0.0350 (11)	0.0002 (8)	0.0226 (9)	−0.0001 (8)
C2	0.0214 (9)	0.0189 (9)	0.0260 (9)	0.0025 (7)	0.0129 (7)	0.0009 (7)
C3	0.0207 (9)	0.0161 (8)	0.0237 (9)	0.0028 (6)	0.0109 (7)	0.0020 (6)
C4	0.0296 (10)	0.0159 (8)	0.0288 (10)	−0.0009 (7)	0.0157 (8)	−0.0008 (7)
C5	0.0331 (11)	0.0219 (9)	0.0285 (10)	0.0009 (8)	0.0176 (9)	−0.0028 (7)
C6	0.0213 (9)	0.0263 (9)	0.0239 (9)	0.0019 (7)	0.0119 (8)	0.0050 (7)
C7	0.0239 (9)	0.0204 (8)	0.0301 (10)	−0.0038 (7)	0.0129 (8)	−0.0001 (7)
C8	0.0257 (9)	0.0182 (8)	0.0281 (10)	−0.0014 (7)	0.0138 (8)	−0.0027 (7)

### Geometric parameters (Å, º)

**Table:**

Br1—C6	1.8906 (19)	C3—C8	1.394 (2)
O1—C2	1.226 (2)	C4—C5	1.380 (3)
N1—C2	1.347 (2)	C4—H4	0.9500
N1—C3	1.401 (2)	C5—C6	1.384 (3)
N1—H1	0.8922	C5—H5	0.9500
C1—C2	1.501 (3)	C6—C7	1.384 (3)
C1—H1A	0.9800	C7—C8	1.377 (3)
C1—H1B	0.9800	C7—H7	0.9500
C1—H1C	0.9800	C8—H8	0.9500
C3—C4	1.390 (2)		
			
C2—N1—C3	127.45 (15)	C5—C4—C3	120.52 (17)
C2—N1—H1	117.0	C5—C4—H4	119.7
C3—N1—H1	115.1	C3—C4—H4	119.7
C2—C1—H1A	109.5	C4—C5—C6	119.17 (17)
C2—C1—H1B	109.5	C4—C5—H5	120.4
H1A—C1—H1B	109.5	C6—C5—H5	120.4
C2—C1—H1C	109.5	C5—C6—C7	121.18 (18)
H1A—C1—H1C	109.5	C5—C6—Br1	119.76 (15)
H1B—C1—H1C	109.5	C7—C6—Br1	119.06 (15)
O1—C2—N1	123.83 (17)	C8—C7—C6	119.33 (18)
O1—C2—C1	121.58 (17)	C8—C7—H7	120.3
N1—C2—C1	114.59 (16)	C6—C7—H7	120.3
C4—C3—C8	119.39 (18)	C7—C8—C3	120.39 (18)
C4—C3—N1	117.09 (16)	C7—C8—H8	119.8
C8—C3—N1	123.46 (17)	C3—C8—H8	119.8
			
C3—N1—C2—O1	−3.1 (3)	C4—C5—C6—C7	−0.7 (3)
C3—N1—C2—C1	176.68 (18)	C4—C5—C6—Br1	179.88 (15)
C2—N1—C3—C4	164.06 (18)	C5—C6—C7—C8	0.0 (3)
C2—N1—C3—C8	−18.6 (3)	Br1—C6—C7—C8	179.37 (15)
C8—C3—C4—C5	0.8 (3)	C6—C7—C8—C3	1.2 (3)
N1—C3—C4—C5	178.23 (17)	C4—C3—C8—C7	−1.6 (3)
C3—C4—C5—C6	0.3 (3)	N1—C3—C8—C7	−178.81 (18)

### Hydrogen-bond geometry (Å, º)

Cg1 is the centroid of the C3–C8 ring.

**Table:**

*D*—H···*A*	*D*—H	H···*A*	*D*···*A*	*D*—H···*A*
N1—H1···O1^i^	0.89	2.00	2.885 (2)	174
C1—H1*B*···*Cg*1^ii^	0.98	2.84	3.761 (3)	157

Symmetry codes: (i) −*x*+1, *y*+1/2, −*z*+1/2; (ii) *x*+1, *y*, *z*.
